# Predictors of mosquito bed net use among children under-fives in Ghana: a multilevel analysis of the 2019 malaria indicator survey

**DOI:** 10.1186/s12936-023-04634-y

**Published:** 2023-06-26

**Authors:** Justice Moses K. Aheto, Rahmatu Babah, Maxwell Kwame Dzokoto, Williams Kwarah, Yakubu Alhassan

**Affiliations:** 1 Department of Biostatistics, School of Public Health, College of Health Sciences, University of Ghana, P. O. Box LG13, Legon-Accra, Ghana; 2 WorldPop, School of Geography and Environmental Science, University of Southampton, Southampton, SO17 1BJ UK; 3 College of Public Health, University of South Florida, Tampa, USA

**Keywords:** Under-five, Mosquito, Insecticide treated net, ITN, Utilization, Malaria, Under-five malaria, Predictors, Ghana, Sub-Saharan Africa

## Abstract

**Background:**

Morbidities and mortalities due to malaria can be prevented by the use of insecticide-treated mosquito bed nets (ITN), which has been proven for malaria control and elimination. The purpose of this study was to assess the critical factors that predict the use of ITN among children under-fives in Ghana.

**Methods:**

The study utilized data from the 2019 Ghana Malaria Indicator Survey (GMIS). The outcome variable was mosquito bed net use among children under-fives. To determine critical factors that independently predict ITN use, multilevel multivariable logistic regression was employed using Stata version 16. Odds ratios and associated 95% confidence intervals and p-values were reported. A *p* < 0.05 was used to declare statistical significance.

**Results:**

The overall prevalence of ITN usage was 57.4%. Utilization of bed nets was 66.6% in the rural areas and 43.5% in the urban areas, was highest in the Upper West region (80.6%) even when stratified to rural (82.9%) and urban areas (70.3%) whilst Greater Accra region (30.5%, rural = 41.7%, urban = 28.9%) had the least. The community level multilevel analysis showed that bed net utilization was higher among children in rural areas [AOR = 1.99, 95% CI 1.32–3.01, p = 0.001] and in household with wooden wall materials [AOR = 3.29, 95% CI 1.15–9.40, p = 0.027]. Bed net utilization was however, less for households with 3 + children under-five [AOR = 0.29, 95% CI 0.19–0.46, p < 0.001), 4 years old (AOR = 0.66, 95% CI 00.48–00.92, p = 0.014], without universal access to bed net [AOR = 0.52, 95% CI 0.37–0.73, p < 0.001], those in the Greater Accra [AOR = 0.26, 95% CI 0.13–0.51, p < 0.001], Eastern [AOR = 0.47, 95% CI 0.23–0.95, p = 0.036], Northern [AOR = 0.42, 95% CI 0.20–0.88, p = 0.022], middle [AOR = 0.57, 95% CI 0.35–0.94, p = 0.026] and rich/richest [AOR = 0.51, 95% CI 0.29–0.92, p = 0.025] household wealth quintile. Substantial unobserved household and community level differences in bed net use were found.

**Conclusion:**

This study demonstrates the need to intensify promotion of ITN use to those in urban areas, Greater Accra, Eastern and Northern regions, houses without wooden wall materials, middle and rich/richest households. Interventions should be targeted at older children and households with more under-five children and to ensure full access and use of ITNs among all children under-fives in each household as part of the overall goal of achieving the health-related SDGs.

## Background

Malaria is a life-threatening disease caused by the *Plasmodium* parasite*,* which is transmitted to humans through the bites of infected mosquitoes. The parasite infects red blood cells and multiplies inside the human body, causing symptoms such as fever, headache, muscle pain, and chills. In severe cases, it can lead to anaemia, kidney failure, and even death. Malaria is a major public health problem globally, particularly in sub-Saharan Africa where the majority of malaria cases and deaths occur [1]. Children under 5 years of age and pregnant women are particularly vulnerable to malaria, as they have weaker immune systems and are at higher risk of severe illness and death [2]. Malaria is a leading cause of death among children under 5 years of age, particularly in sub-Saharan Africa. According to the World Health Organization (WHO), malaria caused an estimated 409,000 deaths among children under 5 years old in 2020, accounting for approximately 60% of all malaria deaths globally [3].

In Ghana, malaria is a serious public health issue and the leading cause of morbidity and mortality and responsible for approximately 38% of outpatient visits and 27% of hospital admissions in health facilities, about 49% of under-five deaths and 7% of all admission deaths in the country [4]. Morbidities and mortalities due to malaria can be prevented through a combination of measures, including the use of insecticide-treated mosquito nets, indoor residual spraying, prompt and effective treatment of malaria cases, and access to preventive measures for pregnant women and children [5, 6]. It is important to continue the efforts to control and eliminate malaria and to ensure that no child dies from a preventable and treatable disease.

The use of mosquito nets is an important strategy in reducing the risk of malaria infection, particularly among children under 5 years of age, who are at higher risk of severe illness and death from the disease. Globally, there has been significant progress in increasing access to and use of mosquito nets in recent years, due to efforts by governments, international organizations, and private sector partners. For example, the Global Fund to Fight AIDS, Tuberculosis, and Malaria, and other organizations, have helped to provide millions of insecticide-treated mosquito nets to families in need in low- and middle-income countries. However, despite these efforts, there are still many areas where access to and use of mosquito nets remains a challenge. In some countries, cultural, behavioural, and financial barriers prevent families from using the nets, and in others, the nets may not be of high quality (e.g., heat and associated discomfort) or may not be used consistently [2, 7–11].

In Ghana, the use of mosquito nets is an important strategy in reducing the risk of malaria infection, particularly among children under 5 years of age who are at higher risk of severe illness and death from the disease. The National Malaria Control Programme of Ghana has implemented several initiatives to distribute mosquito nets to families and to increase awareness of the importance of using them. However, despite these efforts, there are still systemic challenges to increasing access to and use of mosquito nets in Ghana. It is possible that some households and/or communities may not have access to high-quality nets, or may not use them consistently due to cultural, behavioural, or financial barriers [11].

Majority of the studies conducted in sub-Saharan Africa countries, such as Ghana, to identify factors associated with bed net use focused only on having at least a child under-five in a household who slept under bed net the previous night of the interview [12–14], neglecting the benefits of identifying factors that are mainly predictive of the outcome where all under-five children in each household slept under a bed net. Thus, very little is known about factors associated with bed net use among children where all the children under-fives in each household slept under a bed net. This information is critical to universal health coverage and achieving the health-related SDGs, especially SDG Target 3.3 where one of the aims is to reduce malaria case incidence and malaria mortality rates to at least 90% each by 2030, end the epidemic of malaria in at least 35 countries by 2030, and to prevent a resurgence of malaria in all malaria-free countries. It is worthy of note that Ghana has moved from the control of malaria to the elimination of malaria stage in her ambitious fight against malaria, and this required a universal bed net use in her population, especially among children under-fives who are the hardest hit. Also, the bed net use is a continuous phenomenon, which requires a continuous analysis of bed net use data and its predictors to inform timely and sound policy decision when they are made available. Therefore, this study aims to identify critical factors that predict the use of mosquito nets among children under-fives using the most recent nationwide population-based survey data in Ghana utilizing a novel multilevel analysis approach.

## Methods

### Setting, design and sample

This study analysed data from the 2019 Ghana Malaria Indicator Survey. The Ghana Statistical Service (GSS) conducted the 2010 Population and Housing Census (PHC), which served as the sample frame for the 2019 GMIS. The ten regional borders established by the 2010 PHC served as the basis for the sampling frame for the 2019 GMIS. The GMIS is a nationally representative cross-sectional household survey used to gather data on population-based malaria indicators for the country, for urban and rural areas for each of the ten (10) administrative regions as Western, Central, Greater Accra, Volta, Eastern, Ashanti, Brong Ahafo, Northern, Upper East, and Upper West [15].

The sampling procedure was multistage. The sampling frame was stratified into rural and urban areas, creating 20 strata. In the first stage, 200 EAs (97 in urban areas and 103 in rural regions) were chosen independently in each sampling stratum with a probability proportional to the size of the EAs. At each of the lower administrative levels in the second stage, implicit stratification with proportional allocation was accomplished by first sorting the sampling frame within each sampling stratum. About 30 households were selected from each stratum resulting in a sample size of 6000 households overall. The study focused on the dataset for children, comprising about 1,876 women in total aged 15–49 years, and extracted complete data on 2372 children for the study.

### Outcome variable

The outcome variable of interest in this study was children under 5 years who slept under mosquito bed net the previous night of the interview from the household questionnaire. The question asked during the survey was ‘children under 5 slept under mosquito bed net last night’ with responses (1) no, (2) some children, (3) all children, and (4) no net in household. This was recategorized as a binary outcome with responses no or some children slept under the bed net as “0” and all children slept under bed net as “1” because the focus of this study is on under-fives children in a household who slept under bed net the previous night of the interview, and the study aim to identify the factors associated with the probability of all children under-fives in a given household sleeping under bed net compared to where none or only some of them slept under bed net. Note that a response to this question in a given household will be shared by all children under-fives residing in that household. Unlike majority of the previous studies that analysed at least one child sleeping under bed net in household, this novel classification of ‘no or some children’ and ‘all children’ sleeping under bed nets in this study is more informative and critical to public health because its directly predicts the probability of universal usage of bed nets and its associated critical factors to directly support evidenced based data-driven malaria policy decisions and programme implementation relating to universal bed net use and malaria control and elimination efforts. No or some children who slept under bed net (coded as “0”) was used as a reference in the single level and the multilevel logistic regression analyses. Responses that indicated household had no bed net (n = 396) were excluded from the analysis because the focus of the study is about households with at least one bed net given the outcome under study.

### Covariates

The study considered socio-demographic, economic and community level factors as covariates in the analysis. The factors include region and place of residence, water sources, sanitation facilities, wealth index, gender of household head, the age of household head, respondent educational level, respondents’ age, parity of mother, mother current pregnancy status, type of bed net, availability of bed net, respondent slept under bed net, respondent has health insurance, number of children under 5 in the household, sex of child, the age of child, anaemia status of child, the child had a fever in past 2 weeks, multiple or single births, seen or heard malaria messages on various media, seen or heard messages about sleeping under bed net, how household protect themselves from malaria, heard of malaria vaccine, and would parent/guardian allow child to take malaria vaccine, universal access to bed net (i.e., ≤ 2 persons per net). The study also considered number of nets available in a household and household size, but these were removed in the models because of multicollinearity leading to non-convergence problems. This is expected because both were used to estimate the universal access indicators already included in the model resulting in high correlation between them. However, the universal access to bed nets takes into account the number of nets available in a household and the household size, and thus more informative and useful compared to the other two.

The wealth index was generated in the dataset by assigning scores to households based on the quantity and kind of consumer goods they own, including the television and a car. Other factors were sources of drinking water, the availability of toilets, the type of flooring, and roofing. Principal component analysis was then used to create the scores and each household assigned their respective scores. The overall distribution was split into five categories each representing 20 percent of the population.

### Statistical analysis

Both descriptive and inferential statistical analyses were performed. The prevalence and corresponding 95% confidence intervals of both utilization and non-utilization of bed nets were estimated across the various observed characteristics in the study. The Design effect adjusted chi-square test was used to assess the bivariate association between bed net use and the various characteristics. Quantum Geographic Information System (QGIS) version 3.28.2 was used to generate a choropleth map of the utilization of bed net use among children at the regional level and at rural–urban residence stratified within regions as well as at community (cluster) level.

The binary logistic regression model was used to assess the factors associated with utilization of bed nets fitting 3 different multivariable models. The first model was at single model only considering unobserved variation at the child level, the second was a household multilevel model adjusting for unobserved variation at the household level and the third was a community multilevel model adjusting for unobserved variations at the community level. The random intercept binary multilevel model formulation in which the study allowed for clustering (i.e., under-five child nested within a household or community) in the data. Let *P*_*ij*_ be the probability that an under-five child *i* reside in household *j,*$\frac{{P}_{ij}}{1-{P}_{ij}}$ is the odds of bed net use by an under-five child* i* residing in household *j*. The model formulation is given as:

1 $$ {\text{log}}\left( {\frac{{P_{ij} }}{{1 - P_{ij} }}} \right) = \beta_{0} + d\left( {x_{ij} } \right)^{^{\prime}} \beta + v_{0j} $$

where ${\beta }_{0}$ is the overall mean probability of bed net use shared by all under-five children in the households, *d(.)* is a vector of risk factors, *β* is a vector of regression coefficients, ${v}_{0j}$ is the unobserved household-level residual assumed to follow a normal distribution with mean zero (0) and variance ${\sigma }_{v}^{2}$. The level one residual was assumed to follow the standard logistic distribution function with variance $\frac{{\pi }^{2}}{3}$. Secondly, we fitted a separate random intercept multilevel model that accounted for both child and community level random effects and compared this to the child and household level random effects model presented in Eq. (1). Using Eq. (1) and the one for the community level random effect, the study estimated the corresponding intra-household (*ICC*_*h*_) and intra-community (*ICC*_*c*_) correlation coefficient which coincide with the variance partitioning coefficient (VPC) to be:

2 $$ VPC_{h} = \frac{{\sigma_{v}^{2} }}{{\sigma_{v}^{2} + \frac{{\pi^{2} }}{3}}} \times \,\,100\,\, {\text{and}} \,VPC_{c} = \frac{{\sigma_{c}^{2} }}{{\sigma_{c}^{2} + \frac{{\pi^{2} }}{3}}} \times 100 $$

The study adjusted accordingly to account for the influence of significant covariates on the outcome measure. Prior to fitting the final model, we tested for multicollinearity and discovered no collinearity (mean VIF less than 10). To examine how well the model fits the data, the Hosmer–Lemeshow goodness of fit test was conducted. Sampling weight were employed in all analyses to account for the complex survey methodology utilized in the GMIS. The three multivariable models were assessed using the Akaike information criteria (AIC), the Bayesian information criteria (BIC) and the area under the operating characteristics curve (AUROCC). Odd ratios were presented, along with the 95% confidence intervals (CIs). All analyses were done using Stata version 16.1 (StataCorp, College Station, Texas, USA). A *p-value* < 0.05 was used to declare statistical significance.

### Ethical consideration

This study used data from the publicly available MEASURE DHS program data sets with no direct contact with the respondents. The data did not include information that can directly or indirectly be linked to those who participated in the study. Permission was granted by DHS MEASURE Program to use the 2019 GMIS data for the study. The data is freely available after a simple, registration-access request at the link https://dhsprogram.com/data/dataset_admin/index.cfm. The protocol for the 2019 GMIS was approved by the Ghana Health Service Ethical Review Committee and ICF’s Institutional Review Board [15].

### The role of the funding source

The present study did not receive any support from any funding source. Also, the funders of the original survey played no role in the design, data collection, analysis, interpretation, writing of the manuscript, and the decision to submit this manuscript. The corresponding author confirm that he has full access to all the data in this study and accept responsibility to submit for publication.

## Results

### Background characteristics and bivariate analysis

Table 1 shows the bivariate analysis of utilization of bed nets among children under-five years across the various socio-demographic characteristics. Among the 2372 children in the study, 51.3% were males, 21.7% were infants. More than a fifth (22.7%) of the caregivers had formal education with 4.7% having higher than secondary education, a fifth of the caregivers were below 25 years of age with majority of 60.5% having access to health insurance. Majority (61.2%) were residing in rural areas, access to improve water was 36.7%. Majority (28.2%) of the children belonged to households with 2 bed nets while 27% belonged to households with 4 or more bed nets. Majority (50.4%) of the children belonged to households with household size of 4–6 members and 51.5% of children belong to households without universal access to bed net (i.e., > 2 persons per nets) (Table 1).

**Table 1:** Percentage distribution of children under-five by bed net use and selected background characteristics, and bivariate analysis (n = 2372^a^)

Characteristics	Total	Bed net use		Design effect adjusted chi-square test
	N = 2372	No use	Used	
	%^C^	% [95% CI]^R^	% [95% CI]^R^	
Overall	100.0	42.6 [38.9, 46.5]	57.4 [53.5, 61.1]	
Number of under 5 children in household				χ2 = 11.75, p < 0.001
1	40.5	39.2 [34.6, 44.0]	60.8 [56.0, 65.4]	
2	38.8	37.9 [33.5, 42.5]	62.1 [57.5, 66.5]	
3 +	20.7	58.3 [48.9, 67.2]	41.7 [32.8, 51.1]	
Sex of child				χ2 = 1.88, p = 0.172
Male	51.3	44.3 [39.5, 49.3]	55.7 [50.7, 60.5]	
Female	48.7	40.8 [36.7, 45.1]	59.2 [54.9, 63.3]	
Child’s age				χ2 = 1.54, p = 0.199
0	21.7	42.6 [37.5, 47.8]	57.4 [52.2, 62.5]	
1	21.4	37.7 [32.6, 43.0]	62.3 [57.0, 67.4]	
2	21.1	42.7 [35.8, 49.8]	57.3 [50.2, 64.2]	
3	18.4	43.4 [36.3, 50.8]	56.6 [49.2, 63.7]	
4	17.4	47.9 [41.1, 54.8]	52.1 [45.2, 58.9]	
Education				χ2 = 0.67, p = 0.534
No education	22.7	42.4 [32.8, 52.7]	57.6 [47.3, 67.2]	
Primary	21.8	40.0 [34.1, 46.1]	60.0 [53.9, 65.9]	
Secondary	50.8	42.9 [38.0, 47.9]	57.1 [52.1, 62.0]	
Higher	4.7	53.1 [38.6, 67.0]	46.9 [33.0, 61.4]	
Respondents age				χ2 = 0.71, p = 0.539
< 25	21.2	38.8 [31.9, 46.1]	61.2 [53.9, 68.1]	
25–34	50.5	43.7 [38.7, 48.8]	56.3 [51.2, 61.3]	
35–44	25.6	43.0 [36.9, 49.4]	57.0 [50.6, 63.1]	
45 +	2.7	49.2 [32.9, 65.7]	50.8 [34.3, 67.1]	
Region				χ2 = 6.46, p < 0.001
Western	10.5	28.1 [20.0, 38.0]	71.9 [62.0, 80.0]	
Central	7.6	40.2 [31.7, 49.3]	59.8 [50.7, 68.3]	
Greater	11.3	69.5 [60.8, 77.0]	30.5 [23.0, 39.2]	
Volta	12.1	32.1 [23.6, 42.0]	67.9 [58.0, 76.4]	
Eastern	10.4	52.7 [36.9, 68.0]	47.3 [32.0, 63.1]	
Ashanti	16.1	41.7 [31.6, 52.6]	58.3 [47.4, 68.4]	
Brong Ahafo	9.3	33.4 [22.8, 46.0]	66.6 [54.0, 77.2]	
Northern	14.9	50.1 [41.9, 58.3]	49.9 [41.7, 58.1]	
Upper East	4.6	31.9 [22.3, 43.4]	68.1 [56.6, 77.7]	
Upper West	3.1	19.4 [14.4, 25.6]	80.6 [74.4, 85.6]	
Residence				χ2 = 31.98, p < 0.001
Urban	38.8	56.5 [50.3, 62.5]	43.5 [37.5, 49.7]	
Rural	61.2	33.8 [29.2, 38.8]	66.2 [61.2, 70.8]	
Religion				χ2 = 1.76, p = 0.177
Christian	74.9	42.3 [37.9, 46.8]	57.7 [53.2, 62.1]	
Islam	20.9	46.4 [38.6, 54.4]	53.6 [45.6, 61.4]	
Other	4.2	30.1 [19.6, 43.2]	69.9 [56.8, 80.4]	
Ethnicity				χ2 = 1.04, p = 0.370
Akan	40.3	44.0 [38.6, 49.6]	56.0 [50.4, 61.4]	
Ewe	16	37.2 [29.7, 45.5]	62.8 [54.5, 70.3]	
Mole-Dag	21.4	39.7 [32.1, 47.9]	60.3 [52.1, 67.9]	
Others	22.3	46.7 [37.6, 56.1]	53.3 [43.9, 62.4]	
Wealth				χ2 = 18.31, p < 0.001
Poor/poorest	47.2	32.4 [27.2, 38.2]	67.6 [61.8, 72.8]	
Middle	20.5	43.1 [36.8, 49.6]	56.9 [50.4, 63.2]	
Rich/richest	32.3	57.2 [50.5, 63.7]	42.8 [36.3, 49.5]	
Access to water				χ2 = 11.16, p = 0.001
Improved	36.7	50.5 [44.0, 57.0]	49.5 [43.0, 56.0]	
Unimproved	63.3	38.1 [33.9, 42.5]	61.9 [57.5, 66.1]	
Access to toilet facility				χ2 = 10.96, p = 0.001
Improved	37.8	34.4 [28.8, 40.4]	65.6 [59.6, 71.2]	
Unimproved	62.2	47.6 [42.6, 52.7]	52.4 [47.3, 57.4]	
Main floor material				χ2 = 3.23, p = 0.042
Cement	59.6	39.6 [35.1, 44.3]	60.4 [55.7, 64.9]	
Floor carpet	24	50.5 [43.1, 57.8]	49.5 [42.2, 56.9]	
Other	16.4	42.1 [33.5, 51.3]	57.9 [48.7, 66.5]	
Main wall material				χ2 = 4.34, p = 0.017
Cement blocks/bricks	77.3	45.2 [41.0, 49.5]	54.8 [50.5, 59.0]	
Wood	3.4	29.1 [17.9, 43.5]	70.9 [56.5, 82.1]	
Other	19.2	34.5 [26.6, 43.4]	65.5 [56.6, 73.4]	
Main roof material				χ2 = 0.01, p = 0.923
Other	10	43.0 [34.6, 52.0]	57.0 [48.0, 65.4]	
Zinc/Alu	90	42.6 [38.7, 46.6]	57.4 [53.4, 61.3]	
Sex of household head				χ2 = 0.28, p = 0.600
Female	70.6	42.1 [38.1, 46.2]	57.9 [53.8, 61.9]	
Male	29.4	43.9 [37.5, 50.5]	56.1 [49.5, 62.5]	
Age of household head				χ2 = 1.69, p = 0.170
< 25	4.1	26.8 [15.3, 42.5]	73.2 [57.5, 84.7]	
25–34	30.9	41.6 [35.4, 48.1]	58.4 [51.9, 64.6]	
35–44	30.7	42.8 [36.2, 49.6]	57.2 [50.4, 63.8]	
45 +	34.3	45.3 [40.4, 50.4]	54.7 [49.6, 59.6]	
Literacy of respondents				χ2 = 3.37, p = 0.068
Cannot read	53.2	39.6 [34.7, 44.7]	60.4 [55.3, 65.3]	
Can read	46.8	46.1 [40.9, 51.4]	53.9 [48.6, 59.1]	
Covered by health insurance				χ2 = 0.28, p = 0.751
No	45	43.7 [38.6, 48.9]	56.3 [51.1, 61.4]	
Yes	55	41.7 [37.3, 46.3]	58.3 [53.7, 62.7]	
Heard malaria messages				χ2 = 0.47, p = 0.495
No	40.4	41.2 [35.9, 46.8]	58.8 [53.2, 64.1]	
Yes	59.6	43.6 [38.9, 48.3]	56.4 [51.7, 61.1]	
Heard malaria messages on radio				χ2 = 1.31, p = 0.254
No	76.2	41.5 [37.5, 45.7]	58.5 [54.3, 62.5]	
Yes	23.8	46.1 [39.0, 53.4]	53.9 [46.6, 61.0]	
Heard malaria messages on tv				χ2 = 7.76, p = 0.006
No	63.7	39.2 [34.9, 43.7]	60.8 [56.3, 65.1]	
Yes	36.3	48.6 [43.0, 54.3]	51.4 [45.7, 57.0]	
Number of mosquito nets in household				χ2 = 12.30, p < 0.001
1 net	21.6	58.3 [51.1, 65.0]	41.7 [35.0, 48.9]	
2 nets	28.2	34.4 [29.7, 39.5]	65.6 [60.5,70.3]	
3 nets	23.2	44.1 [37.2, 51.3]	55.9 [48.7, 62.8]	
4 + nets	27	37.4 [31.9, 43.4]	62.6 [56.6, 68.1]	
Household size				χ2 = 9.42, p < 0.001
2–3 members	14.5	37.0 [30.0, 44.7]	63.0 [55.3, 70.0]	
4–6 members	50.4	41.0 [35.9, 46.3]	59.0 [53.7, 64.1]	
7–9 members	24.2	38.8 [33.3, 44.6]	61.2 [55.4, 66.7]	
10 + members	10.8	66.5 [55.1, 76.3]	33.5 [23.7, 44.9]	
Number of persons per mosquito net				χ2 = 19.35, p < 0.001
≤ 2 persons per net	48.5	35.1 [30.4, 40.2]	64.9 [59.8, 69.6]	
> 2 persons per net	51.5	49.7 [44.7, 54.7]	50.3 [45.3, 55.3]	

^*C*^ Column percentage, ^*R*^ Row percentage, *CI* confidence interval

^a^Restricted to households with at least one bed net

### Use of bed nets among children in the household

**Fig. 1:**
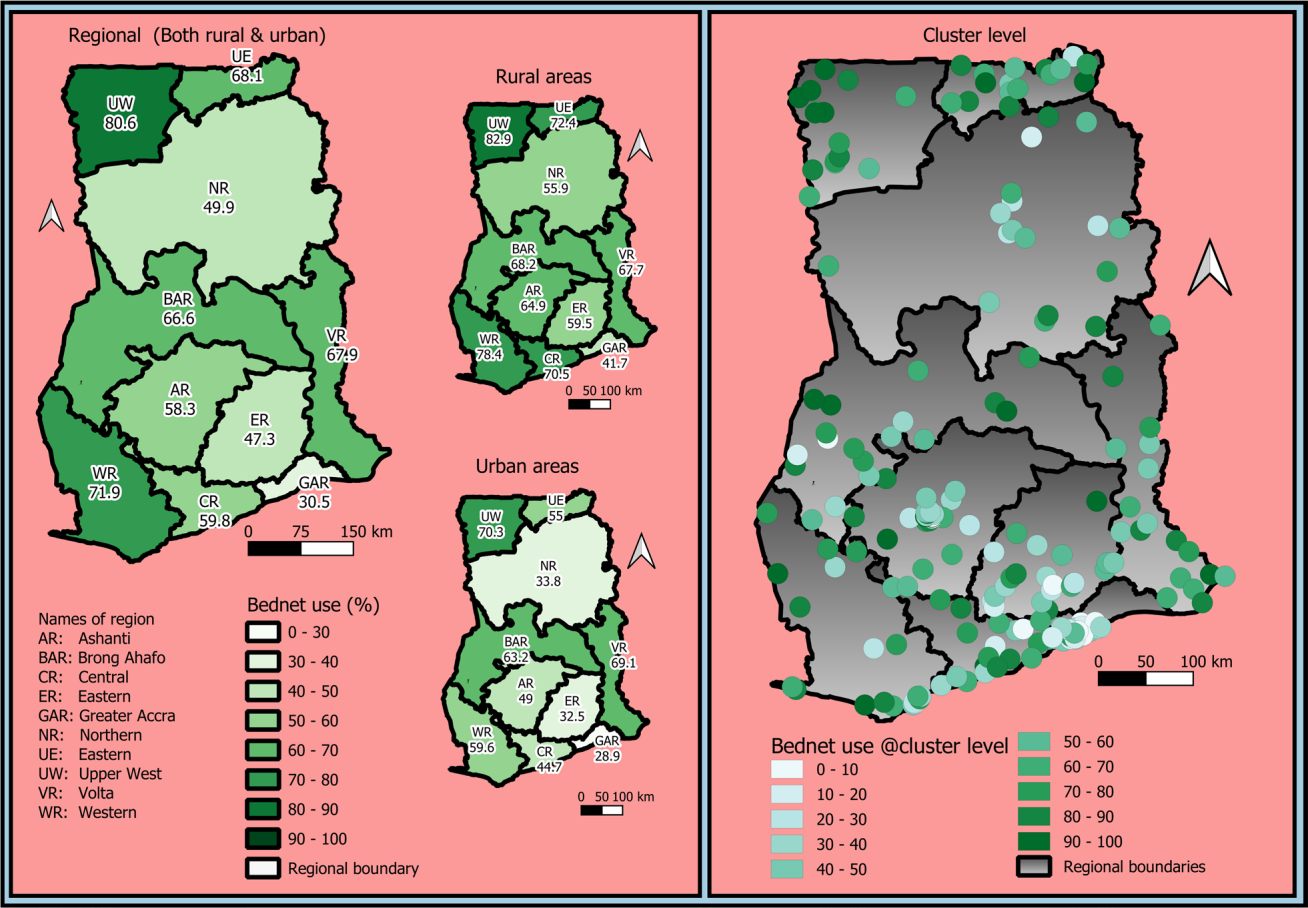
Utilization of bed nets among children under-five years at the regional, Rural, Urban and cluster/community level. Note that the results here were restricted to households with at least one bed net

Among the 2372 children, 57.4% slept under bed nets. The use of bed net was 66.2% in rural areas and 43.5% in urban areas (Table 1). The Upper West region (80.6%) recorded the highest bed net utilization whilst the Greater Accra region recorded the lowest (30.5%). In terms of the stratification of rural and urban areas within regions, bed net utilization was highest in the rural areas of the Upper West region (82.9%) and least in the Greater Accra region (41.7%). Also, bed net utilization was highest in the urban areas of the Upper West region (70.3%) and least in the urban areas of the Greater Accra (28.9%), Eastern (32.5%) and the Northern (33.8%) regions. Finally, the study presents. The geospatial map of bed net utilization among children under-five years at the cluster level (Fig. 1).

### Binary logistic regression model of factors associated with utilization of bed net among children under-fives

**Fig. 2:**
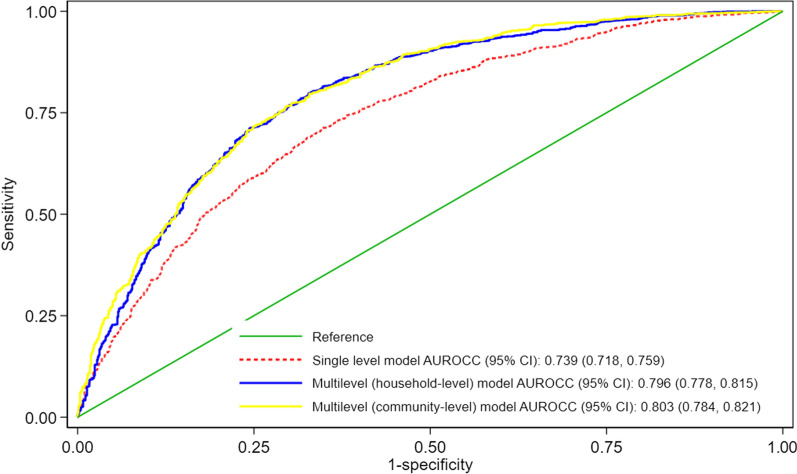
Area Under Operating Characteristics Curve for correctly predicting bed net use among under-five children in households from the single and multilevel multivariable binary logistic regression models. Note that the results here were restricted to households with at least one bed net

The single-level multivariable logistic regression identified sex of child, three or more under-five in household, Greater Accra, Eastern, Northern and Upper East regions, place of residence, wealth, access to toilet facility, wall material, heard malaria message on tv as significant predictors of bed net use among under-five children. The multilevel model provided a good fit to the data when compared with the single-level logistic regression model (Table 2). Figure 2 showed the predictive ability of the fitted models to correctly predict mosquito bed net use among this group of children under-five in Ghana. The predictive ability of the single level model was 74% (95% CI 72, 76), 80% (95% CI 78, 82) for the household level multilevel model and 80% (95% CI 78, 82) for the community level multilevel model. The community multilevel model provided slightly higher predictive ability than the household multilevel model (Fig. 2). Also, the community multilevel was a better model using both the AIC and the BIC model evaluation metrics. Thus, the community level random intercept model was prefered to the household level model hence the interpretations and conclusion are based on the community level random intercept multilevel model (Table 2).

**Table 2:** Factors influencing bed net ownership and use among under-five children from a multilevel model (N = 2372^a^)

Characteristics			Adjusted model					
	Unadjusted model		Single level model		Multilevel (household)		Multilevel (community)	
	COR [95% CI]	P-value	AOR [95% CI]	P-value	AOR [95% CI]	P-value	AOR [95% CI]	P-value
Number of under 5 children in household								
1	1.00 [reference]		1.00 [reference]		1.00 [reference]		1.00 [reference]	
2	1.06 [0.84, 1.33]	0.644	0.84 [0.66, 1.09]	0.187	0.82 [0.59, 1.15]	0.261	0.78 [0.61, 0.99]	0.045
3 +	0.46 [0.31, 0.68]	< 0.001	0.37 [0.27, 0.51]	< 0.001	0.38 [0.23, 0.64]	< 0.001	0.34 [0.21, 0.54]	< 0.001
Sex of child								
Male	1.00 [reference]		1.00 [reference]		1.00 [reference]		1.00 [reference]	
Female	1.15 [0.94, 1.42]	0.172	1.23 [0.99, 1.53]	0.056	1.23 [0.99, 1.53]	0.064	1.20 [0.95, 1.51]	0.121
Child’s age								
0	1.00 [reference]		1.00 [reference]		1.00 [reference]		1.00 [reference]	
1	1.23 [0.96, 1.58]	0.108	1.11 [0.79, 1.57]	0.533	1.17 [0.83, 1.64]	0.378	1.08 [0.81, 1.43]	0.611
2	1.00 [0.72, 1.37]	0.980	0.87 [0.61, 1.25]	0.459	0.89 [0.65, 1.21]	0.460	0.86 [0.60, 1.22]	0.393
3	0.97 [0.71, 1.32]	0.830	0.91 [0.65, 1.27]	0.585	0.90 [0.65, 1.24]	0.516	0.88 [0.64, 1.20]	0.414
4	0.81 [0.60, 1.08]	0.147	0.69 [0.49, 0.99]	0.042	0.70 [0.50, 0.98]	0.035	0.64 [0.46, 0.89]	0.008
Education								
No education	1.00 [reference]		1.00 [reference]		1.00 [reference]		1.00 [reference]	
Primary	1.11 [0.67, 1.84]	0.696	1.12 [0.77, 1.63]	0.556	1.08 [0.65, 1.78]	0.775	1.07 [0.59, 1.94]	0.821
Secondary	0.98 [0.62, 1.56]	0.933	1.26 [0.88, 1.80]	0.202	1.20 [0.73, 1.97]	0.477	1.29 [0.72, 2.32]	0.385
Higher	0.65 [0.30, 1.40]	0.270	1.34 [0.74, 2.44]	0.335	1.29 [0.53, 3.16]	0.581	1.26 [0.54, 2.90]	0.592
Respondents age								
< 25	1.00 [reference]		1.00 [reference]		1.00 [reference]		1.00 [reference]	
25–34	0.82 [0.57, 1.17]	0.262	1.13 [0.82, 1.55]	0.449	1.07 [0.71, 1.60]	0.759	1.16 [0.72, 1.88]	0.534
35–44	0.84 [0.56, 1.26]	0.390	1.25 [0.87, 1.80]	0.234	1.23 [0.76, 1.97]	0.397	1.30 [0.72, 2.36]	0.389
45 +	0.65 [0.32, 1.35]	0.249	0.98 [0.53, 1.80]	0.938	1.15 [0.50, 2.64]	0.735	1.01 [0.44, 2.35]	0.978
Region								
Western	1.00 [reference]		1.00 [reference]		1.00 [reference]		1.00 [reference]	
Central	0.58 [0.33, 1.04]	0.068	0.68 [0.44, 1.05]	0.084	0.68 [0.32, 1.42]	0.306	0.78 [0.40, 1.52]	0.468
Greater Accra	0.17 [0.09, 0.31]	< 0.001	0.27 [0.16, 0.45]	< 0.001	0.22 [0.11, 0.47]	< 0.001	0.27 [0.14, 0.53]	< 0.001
Volta	0.83 [0.44, 1.54]	0.544	0.71 [0.42, 1.22]	0.217	0.69 [0.30, 1.57]	0.380	0.97 [0.50, 1.89]	0.923
Eastern	0.35 [0.16, 0.77]	0.009	0.44 [0.28, 0.70]	< 0.001	0.42 [0.21, 0.85]	0.015	0.48 [0.24, 0.96]	0.039
Ashanti	0.55 [0.29, 1.02]	0.058	0.62 [0.39, 0.96]	0.034	0.57 [0.30, 1.08]	0.086	0.69 [0.36, 1.34]	0.276
Brong Ahafo	0.78 [0.39, 1.56]	0.477	0.74 [0.47, 1.16]	0.190	0.68 [0.35, 1.30]	0.240	0.96 [0.46, 2.01]	0.910
Northern	0.39 [0.22, 0.68]	0.001	0.39 [0.24, 0.63]	< 0.001	0.34 [0.16, 0.73]	0.005	0.45 [0.21, 0.97]	0.041
Upper east	0.83 [0.43, 1.62]	0.590	0.61 [0.37, 0.99]	0.043	0.59 [0.27, 1.30]	0.190	0.72 [0.36, 1.44]	0.349
Upper west	1.63 [0.91, 2.89]	0.097	1.40 [0.83, 2.35]	0.205	1.58 [0.75, 3.35]	0.229	1.57 [0.77, 3.21]	0.214
Residence								
Urban	1.00 [reference]		1.00 [reference]		1.00 [reference]		1.00 [reference]	
Rural	2.54 [1.83, 3.53]	< 0.001	1.57 [1.20, 2.06]	0.001	1.58 [1.10, 2.26]	0.012	1.88 [1.24, 2.84]	0.003
Religion								
Christian	1.00 [reference]		1.00 [reference]		1.00 [reference]		1.00 [reference]	
Islam	0.85 [0.59, 1.23]	0.378	0.96 [0.69, 1.36]	0.837	0.90 [0.58, 1.39]	0.622	1.19 [0.77, 1.86]	0.434
Other	1.70 [0.92, 3.12]	0.088	1.46 [0.77, 2.76]	0.243	1.54 [0.66, 3.63]	0.318	1.45 [0.74, 2.82]	0.277
Wealth								
Poorest/poor	1.00 [reference]		1.00 [reference]		1.00 [reference]		1.00 [reference]	
Middle	0.63 [0.45, 0.89]	0.008	0.52 [0.36, 0.74]	< 0.001	0.44 [0.26, 0.76]	0.003	0.53 [0.32, 0.88]	0.014
Rich/richest	0.36 [0.24, 0.53]	< 0.001	0.40 [0.26, 0.60]	< 0.001	0.34 [0.19, 0.61]	< 0.001	0.47 [0.26, 0.85]	0.013
Access to water								
Improved	1.00 [reference]		1.00 [reference]		1.00 [reference]		1.00 [reference]	
Unimproved	1.66 [1.23, 2.24]	0.001	1.22 [0.93, 1.59]	0.150	1.26 [0.89, 1.78]	0.199	1.35 [0.94, 1.94]	0.102
Access to toilet facility								
Improved	1.00 [reference]		1.00 [reference]		1.00 [reference]		1.00 [reference]	
Unimproved	0.58 [0.41, 0.80]	0.001	0.78 [0.61, 1.01]	0.056	0.77 [0.52, 1.13]	0.180	0.72 [0.50, 1.05]	0.088
Main floor material								
Cement	1.00 [reference]		1.00 [reference]		1.00 [reference]		1.00 [reference]	
Floor carpet	0.64 [0.46, 0.89]	0.009	0.96 [0.70, 1.31]	0.796	0.94 [0.60, 1.48]	0.805	0.87 [0.54, 1.41]	0.582
Other	0.90 [0.59, 1.37]	0.629	0.93 [0.69, 1.27]	0.661	0.91 [0.61, 1.37]	0.651	0.91 [0.57, 1.45]	0.696
Main wall material								
Cement blocks/bricks	1.00 [reference]		1.00 [reference]		1.00 [reference]		1.00 [reference]	
Wood	2.02 [1.05, 3.87]	0.035	2.85 [1.34, 6.05]	0.006	2.52 [0.85, 7.47]	0.095	3.21 [1.18, 8.74]	0.022
Other	1.57 [1.04, 2.36]	0.033	1.16 [0.86, 1.58]	0.331	1.17 [0.73, 1.88]	0.504	1.07 [0.64, 1.79]	0.807
Age of household head								
< 25	1.00 [reference]		1.00 [reference]		1.00 [reference]		1.00 [reference]	
25–34	0.51 [0.24, 1.11]	0.090	0.70 [0.37, 1.30]	0.257	0.80 [0.34, 1.88]	0.605	0.62 [0.23, 1.64]	0.333
35–44	0.49 [0.22, 1.09]	0.078	0.72 [0.38, 1.36]	0.313	0.77 [0.31, 1.88]	0.562	0.59 [0.19, 1.82]	0.358
45 +	0.44 [0.21, 0.92]	0.029	0.55 [0.30, 1.01]	0.055	0.59 [0.25, 1.41]	0.233	0.47 [0.18, 1.23]	0.122
Covered by health insurance								
No	1.00 [reference]		1.00 [reference]		1.00 [reference]		1.00 [reference]	
Yes	1.08 [0.85, 1.38]	0.515	1.18 [0.94, 1.48]	0.157	1.13 [0.82, 1.56]	0.442	1.24 [0.94, 1.63]	0.136
Heard malaria messages on radio								
No	1.00 [reference]		1.00 [reference]		1.00 [reference]		1.00 [reference]	
Yes	0.83 [0.60, 1.14]	0.255	0.83 [0.63, 1.08]	0.161	0.80 [0.58, 1.12]	0.202	0.92 [0.66, 1.27]	0.605
Heard malaria messages on TV								
No	1.00 [reference]		1.00 [reference]		1.00 [reference]		1.00 [reference]	
Yes	0.68 [0.52, 0.89]	0.006	1.16 [0.89, 1.52]	0.270	1.28 [0.90, 1.82]	0.162	1.23 [0.87, 1.73]	0.237
Number of persons per mosquito net								
≤ 2 persons per net	1.00 [reference]		1.00 [reference]		1.00 [reference]		1.00 [reference]	
> 2 persons per net	0.55 [0.42, 0.72]	< 0.001	0.57 [0.45, 0.72]	< 0.001	0.48 [0.35, 0.64]	< 0.001	0.52 [0.37, 0.73]	< 0.001
Random effect parameter analysis								
Child level variance			π^2^ /3 ≈ 3.29		π^2^ /3 ≈ 3.29		π^2^ /3 ≈ 3.29	
Household level variance (95% CI)					0.63 [0.41, 0.98]		-	
Community level variance (95% CI)					-		0.57 [0.33, 0.96]	
AIC			2732.45		2666.144		2657.185	
BIC			2969.081		2908.547		2899.588	
AUROC (95% CI)			73.9 [71.8, 75.9]		79.7 [77.8, 81.5]		80.3 [78.5, 82.1]	

*COR* crude odds ratio, *AOR* adjusted odds ratio, *CI* Confidence interval, *AIC* Akaike information criteria, *BIC* Bayesian information criteria, *AUROCC* Area under the receiver operating characteristics curve

^a^Restricted to households with at least one bed net

Households with increased number of children had reduced odds of children under-five sleeping under bed nets with a 22% and 66% less odds for households with 2 children [AOR = 0.78, 95% CI 0.61–0.99, p = 0.045] and 3 or more children [AOR = 0.34, 95% CI 0.21–0.54, p < 0.001] respectively relative to 1 child under-five years. Children aged 4 were 36% less likely to use bed net compared to children less than 1 year [AOR = 0.64, 95% CI 0.46–0.89, p = 0.008]. Utilization of bed nets was less among children in Greater Accra [AOR = 0.27, 95% CI 0.14–0.53, p < 0.001]. Eastern [AOR = 0.48, 95% CI 0.24–0.96, p = 0.039] and the Northern region [AOR = 0.45, 95% CI 0.21–0.97, p = 0.030] compared to those in the Western region. Children in rural areas had higher odds of bed net use [AOR = 1.88, 95% CI 1.24–2.84, p = 0.003]. Relative to children from the poorest or poor households, utilization of bed net was 47% and 53% less among children from middle [AOR = 0.53, 95% CI 0.32–0.88, p = 0.014] and rich/richest [AOR = 0.47, 95% CI 0.26–0.85, p = 0.013] households, respectively. Children living in households made of wood wall materials had over 3 times increased odds of using bed net compared to those in cemented wall material houses [AOR = 3.21, 95% CI 1.18–8.74, p = 0.022]. Also, relative to household with universal access to net (≤ 2 person per net), utilization of bed nets among children was significantly 48% less in households without universal access to bed net (> 2 persons per nets) [AOR = 0.52, 95% CI 0.37–0.73, p < 0.001] (Table 2).

From Table 2 and using Eq. (2), we estimated the intra-household- and intra-community correlation coefficient (ICC), which coincided with the variance partitioning coefficient (VPC) to be:

$$ VPC_{h} = \frac{0.63}{{0.63 + 3.29}}\, = \,16.1\% \,\,{\text{and}}\,\,VPC_{c} = \frac{0.57}{{0.57 + 3.29}}\, = \,{14}.{8}\% $$

Thus, about 16% and 15% of differences observed in bed net use among the children can be attributed separately to household, and communities that the children belong to, respectively.

## Discussion

The aim of this study was to determine the predictors of mosquito bed net (ITN) use among children under-five in Ghana using the most recent nationwide population-based survey data. This study is particularly interested in the roles played by the individual households and communities in the utilization or non-utilization of bed net among children under-fives. The study achieved this via multilevel modelling approach by quantifying unobserved household and community level residual effects on bed net use in these children which reflect disparities in household- and community-level bed net use that cannot be explained by the considered child, household, and community level predictors. This is critical to understanding factors that militate or promote the use of bed net at the household and community levels because the health and the general well-being of individuals relies heavily on the households and the communities they belong to due to shared opportunities and risks over their life course [16]. The study found substantial unobserved household- and community-level differences in bed net use, accounting for about 16% and 15% of the differences in bed net use separately at household and community levels respectively, suggesting that the bed net use differ from household to household, and from community to community. Furthermore, the study produced geospatial maps to show geographical differences in crude bed net use in Ghana. The map showed substantial geographical differences in bed net use among children under-fives. Also, region, rural/urban, and clusters level disparities exist. For example, the Upper West region recorded the highest bed net utilization (80.6%) whilst the Greater Accra region recorded the lowest (30.5%). For rural areas within regions, the lowest was recorded in Greater Accra (41.7%) and the highest in Upper West (82.9%) while in the urban areas, it was highest in Upper West (70.3%) and lowest in 28.9%). This is critical to informing policymakers as to where preventive and control efforts can be targeted.

The proportion of all under-fives in a household ITN use among households with at least one bed net in Ghana was 57.4%, a prevalence that is relatively low. The plausible reason for the low usage could be as a result of human behaviour or the information disseminated were not culturally sensitive and also not based on existing positive beliefs and behaviour, and inadequate access to bed nets. Further, the use of other preventive measures such as indoor residual spraying, use of mosquito repellants or sprays, early treatment could explain the low use of the ITNs [17].

This study identified several variables that were associated with utilization of insecticide treated nets. These include age of the child, number of children under-five, residence, region, main wall material, number of persons per bed net (i.e., universal access), and wealth index. Similar to other studies in Africa [12, 18], the older the child, the less likely that child will sleep under bed net. A plausible reason could be that some households have limited number of bed nets and are unable to provide for all children under-fives in the household and priority given to the youngest among all the children. Another explanation that could be given is that as the child grows older, he/she may experience fewer episodes of malaria, and this may give a false sense of protection leading to lower usage of bed net.

Although the findings did not show sex of the child to significantly predict the usage of mosquito bed net in the community multilevel analysis, the results alluded to higher utilization among females. This non-significant association is in line with findings from other studies conducted in Liberia that showed that sex does not make a difference for ITN use among children under-five years [19].

Contrary to other studies that significantly predicted that caregivers with tertiary level of education were more likely to use ITN than those with no formal education [20, 21], this current study did not find level of education as a significant predictor of ITN usage even though the results indicated that increase in the level of education of parents resulted in higher odds of ITN usage. For children whose parents had secondary education, the odds of ITN usage was 1.27 times higher compared to those with no education. The odds increased further among those who had higher education compared to those with no education. Inasmuch as this findings gives credence to Gary Becker’s theory of human capital [22], the implication here is that educated parents tend to understand the benefits of using ITN and are also able to effectively use the net to protect their children. This findings agree with studies conducted in the Gambia, Nigeria, and Guinea [12, 23, 24].

Another significant predictor for ITN use was place of residence. Children from the rural area had higher odds of ITN use compared to those from urban areas. This is similar to previous studies conducted in Africa [25, 26]. This is because the rural areas may have bushes and streams that is conducive for breeding of mosquitoes. This will lead to a high perceived threat of mosquito bite and consequently lead to higher utilization of bed net.

Consistent with previous studies, children from middle and rich/richest households had lower odds of ITN use as compared to those from poorest and poor households [26]. A possible explanation could be that these categories of individuals can afford to send their sick children to hospitals that provide the best of care at a higher cost although this theory seems unlikely. Alternatively, the combination of the findings from four different observed factors in the study, thus children living in the Greater Accra region, those is urban areas, those in a richer household and those living in households with cemented wall materials were less likely to use bed nets. This finding is quite interesting as Greater Accra region is largely urban populated with higher income households compared to other regions. Houses in Greater Accra region are also mostly cemented even in rural areas of the region. People with such characteristics are more likely to live in environment least exposed to mosquitoes or with alternative protections such as building well protected against mosquito entry by fixing proper nets for their windows and doors, good environmental hygiene, regular utilization of indoor insecticide sprays and outdoor fumigation which poor households can hardly afford [26]. This could provide a perceived assurance of protection against mosquito bites, which could result in less utilization of bed nets. However, this finding contradict findings from previous studies that found higher bed net utilization among wealthier households [27, 28]. Further studies in the form of a longitudinal and qualitative analyses are however warranted to establish whether higher wealth index leads to lower or higher usage of ITN.

The study found reduced odds of bed net use among children from households without universal access to bed nets, a finding in the expected direction and consistent with previous studies that found higher utilization among children from households with more access to bed net compared to those with limited access. Thus, inadequate availability of bed nets in households is associated with reduced bed net use, reinforcing the utmost need to increase bed net delivery and access to households according to household size and needs, but not based on a fixed quantity across households in the country [26, 29].

One of the key strengths of the study is that is a nationwide representative population-based survey with internationally approved sound survey methods and quality data on individuals, their households, and communities they belong to, and used the most recent (i.e., 2019) survey data which recorded a 99% response rates for both women, and household interviews. The survey procedures coupled with large samples drawn nationally allow for the findings to be generalized to the population of children under-fives in Ghana and under-fives children in similar other populations worldwide. The modelling approach also allows quantification of unobserved household and community residual effects on under-fives bed net use, thereby providing additional information about the reasons some children from certain households and communities used bed net while other children from other households and communities did not while simultaneously adjusting for predictors of be net use. In addition, sampling weights were adjusted for in all the analyses in the study, making the estimates more reliable and representative of the entire population. The study also produced spatial maps to show geographical differences in bed net use by region, rural/urban, and by clusters to put the findings in context for better understanding. The findings have some limitation which should be considered in interpreting the results. The study utilized data based on a cross-sectional survey design and cannot be used to infer causative effect between the outcome and the predictors. As with all studies, this study did not account for all factors that might predict bed net use in this population of children.

## Conclusion

The findings have direct bearing on achieving SDG Target 3.3 that relates to reduction in malaria case incidence and malaria mortality rates to at least 90% each and to end the epidemic of malaria in at least 35 countries by 2030, and Ghana is currently at the elimination stage of her fight against malaria. The study highlights both child- maternal/household- and community level characteristics to be predictive of bed net use among under-fives population and demonstrated the utmost need to intensify promotion of ITN use to households in urban areas, Greater Accra, Eastern and Northern regions. There is a need to also target older children, houses without wooden wall materials, and middle and rich/richest households with specific interventions to promote ITN use among them. Interventions should also be targeted at older household heads, older children, and households with more under-five children and to ensure full access and use of ITNs among all children under-fives in each household as part of the overall goal of achieving the health-related SDGs. The finding that substantial unobserved household level and community level residual were found warrant further studies to examine other factors not considered in this study which might help explain why all children under-fives in a particular household or community slept under a bed net whilst not all children under-fives from other household or community slept under a bed net.

## Data Availability

The datasets generated and/or analyzed during the current study are available from the Measure DHS Program website http://dhsprogram.com/data/available-datasets.cfm**.**

## Abbreviations

AUROCC: Area under the receiver operating characteristics curve
DHS: Demographic and health surveys
GMIS: Ghana malaria indicator survey
ITN: Insecticide treated net
QGIS: Quantum geographic information system
SDGs: Sustainable development goals
VIF: Variance inflation factor
